# Conservation of Gene Order and Content in the Circular Chromosomes of ‘*Candidatus* Liberibacter asiaticus’ and Other Rhizobiales

**DOI:** 10.1371/journal.pone.0034673

**Published:** 2012-04-04

**Authors:** L. David Kuykendall, Jonathan Y. Shao, John S. Hartung

**Affiliations:** Molecular Plant Pathology Laboratory, Agricultural Research Service, United States Department of Agriculture, Beltsville, Maryland, United States of America; Hospital for Sick Children, Canada

## Abstract

‘*Ca.* Liberibacter asiaticus,’ an insect-vectored, obligate intracellular bacterium associated with citrus-greening disease, also called “HLB," is a member of the *Rhizobiales* along with nitrogen-fixing microsymbionts *Sinorhizobium meliloti* and *Bradyrhizobium japonicum*, plant pathogen *Agrobacterium tumefaciens* and facultative intracellular mammalian pathogen *Bartonella henselae*. Comparative analyses of their circular chromosomes identified 514 orthologous genes shared among all five species. Shared among all five species are 50 identical blocks of microsyntenous orthologous genes (MOGs), containing a total of 283 genes. While retaining highly conserved genomic blocks of microsynteny, divergent evolution, horizontal gene transfer and niche specialization have disrupted macrosynteny among the five circular chromosomes compared. Highly conserved microsyntenous gene clusters help define the Rhizobiales, an order previously defined by 16S RNA gene similarity and herein represented by the three families: *Bartonellaceae, Bradyrhizobiaceae* and *Rhizobiaceae*. Genes without orthologs in the other four species help define individual species. The circular chromosomes of each of the five Rhizobiales species examined had genes lacking orthologs in the other four species. For example, 63 proteins are encoded by genes of ‘*Ca.* Liberibacter asiaticus’ not shared with other members of the Rhizobiales. Of these 63 proteins, 17 have predicted functions related to DNA replication or RNA transcription, and some of these may have roles related to low genomic GC content. An additional 17 proteins have predicted functions relevant to cellular processes, particularly modifications of the cell surface. Seventeen unshared proteins have specific metabolic functions including a pathway to synthesize cholesterol encoded by a seven-gene operon. The remaining 12 proteins encoded by ‘*Ca.* Liberibacter asiaticus’ genes not shared with other *Rhizobiales* are of bacteriophage origin. ‘*Ca.* Liberibacter asiaticus’ shares 11 genes with only *Sinorhizobium meliloti* and 12 genes are shared with only *Bartonella henselae*.

## Introduction

Related bacteria share orthologous proteins and genes. When chromosomes are compared, phylogenetically-related bacteria typically share regions of conserved gene order or microsynteny, formerly called clusters of orthologous genes (COGs). We propose the term MOG for Microsyntenous Orthologous Gene clusters to avoid confusion with clusters of orthologous groups of proteins described in the COG database which is now very widely used to identify and classify proteins and thus to identify and classify orthologous genes by their predicted products. Computational algorithms are available that allow identification of regions containing microsyntenous orthologous genes [1]. The arrangement of MOGs relative to that found in other genomes is revealed by mapping the order of blocks of microsyntenous orthologous genes in two or more genomes under comparison. Blocks of syntenous orthologous gene regions retain a phylogenetic signal over a broad range of bacteria [2]. This approach has been used to discover syntenous regions within the Rhizobiales, where large regions of conserved synteny were observed among free-living members of this order [3]–[4].

‘Huanglongbing’ (HLB), also called ‘citrus greening,’ is the most serious disease of citrus worldwide [5]–[6]. This disease originated in south Asia [7]–[8] but has recently invaded both Brazil [9] and Florida [10], the sources of 90% of the world's supply of orange juice. ‘*Candidatus* Liberibacter asiaticus,’ a member of the α-2 subdivision of the Proteobacteria [11], is widely considered to be the causal agent of the disease, since it is consistently associated with the disease although Koch's postulates have not yet been demonstrated [12]. The pathogen is observed within sieve cells of phloem vessels of infected plant hosts [13] or in the salivary glands of citrus psyllids, the natural vector of the pathogen. ‘*Ca.* Liberibacter asiaticus’ is also routinely detected by PCR assays, most based on the 16S rRNA gene [14]–[15]. The overall distribution within infected citrus trees is highly erratic [16]–[17], although ‘*Ca.* Liberibacter asiaticus’ multiply to very high levels within individual infected cells [18]. In spite of many efforts [19]–[22] ‘*Ca.* Liberibacter asiaticus’ has not been artificially cultured, and thus the bacterium has ‘*Candidatus*’ status.

The complete genome sequence of ‘*Ca.* Liberibacter asiaticus’ was obtained by deep sequencing of DNA obtained from an infected psyllid which was known to have at least 8×10^8^ copies of the ‘*Ca.* Liberibacter asiaticus’ genome [23] and by deep sequencing of laser micro- dissected plant phloem cells that contained the organism [24]. The genome is composed of a circular chromosome of 1.23 Mb without any plasmids. Analysis of the full genomic sequence enabled the placement of ‘*Ca.* Liberibacter asiaticus’ within the family *Rhizobiaceae* of the order *Rhizobiales* [25]–[26] within the class α-*Proteobacteria*.

In order to gain insight into the precise nature of their phylogenetic relationships and adaptations to diverse niches, we compared MOGs in the circular chromosome of ‘*Ca.* Liberibacter asiaticus’ with those encoded by the circular chromosomes in *Sinorhizobium meliloti*, *Bradyrhizobium japonicum* and *Agrobacterium tumefaciens*. These latter bacteria, capable of free-living, nitrogen-fixing symbiotic and pathogenic lifestyles are highly adaptable and thus have large genomes. As bacteria adapt to intracellular parasitism their genomes typically become greatly reduced in size and complexity with a very much lower mol % content of guanine plus cytosine residues [27]. We therefore also compared the genome of ‘*Ca.* Liberibacter asiaticus’ to that of *Bartonella henselae*, a flea-vectored pathogen of cats and, opportunistically, also humans. Although *B. henselae* can be artificially cultured, it has complex nutritional requirements and thus it can be considered a facultative, semi-obligate intracellular pathogen of cats, reproducing in erythrocytes [28] and possessing a severely reduced genome, not much larger than that of ‘*Ca.* Liberibacter asiaticus’ [28]–[29].

The characterization of microsyntenous orthologous genes identified a core gene set that helps to define the order Rhizobiales, members of which have evolved to diverse lifestyles or niches. We also identified an array of genes that were unique to each of the five species compared. These genes encode proteins that contribute to the separate identity of each species. We also identified small sets of genes, twelve or fewer, that were uniquely shared in each pair-wise comparison of circular chromosomes. The genes shared between ‘*Ca.* Liberibacter asiaticus’ and *B. henselae* may be of particular interest as they could provide valuable insight(s) into genes either acquired or conserved as essential for adaptation to a parasitic lifestyle in an intracellular environment. Our analysis provides insight into the genomes of the *Rhizobiales* and identifies features of the ‘*Ca.* Liberibacter asiaticus’ genome that support its adaptation to an intracellular lifestyle in both plant and insect hosts.

## Results

### Identification, characterization and mapping of clusters of orthologous genes conserved among chromosomes of five members of the Rhizobiales

**Figure 1:**
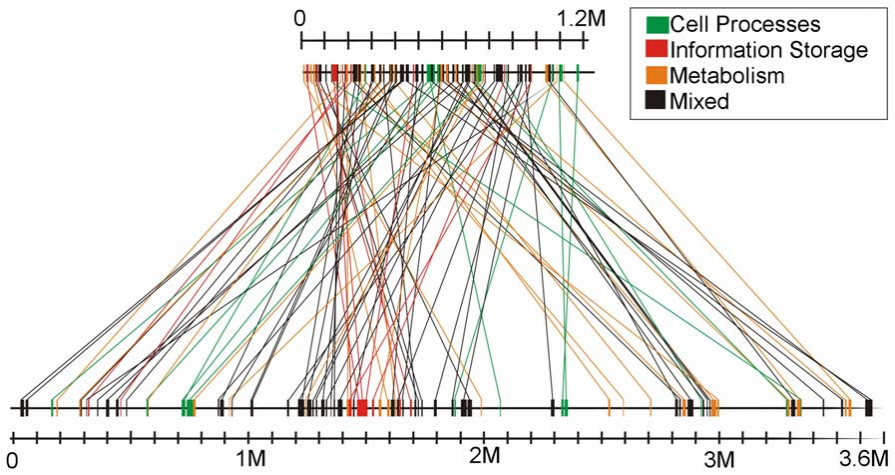
Comparative organization of the circular chromosomes of ‘*Ca.* Liberibacter asiaticus’ and *S. meliloti*. Microsyntenous orthologous genes (MOGs) were identified and plotted on respective chromosomes. Lines connect identical blocks of genes. The upper chromosome is that of ‘*Ca.* Liberibacter asiaticus’, 1.2 Mb. The bottom chromosome is that of chromosome of *Sinorhizobium meliloti* (3.7 Mb).

‘*Ca.* Liberibacter asiaticus’ shares 91, 79, 80 and 66 microsyntenous orthologous gene (MOG) clusters of between 3 and 31 genes with *S. meliloti*, *B. japonicum*, *A. tumefaciens* and *B. henselae*, respectively. Among these MOG clusters, at least 50 identical clusters totaling 283 genes are shared by all five bacterial species (Table S1). Widely different order and relative position of MOGs on the different chromosomes was observed as compared to the ‘*Ca.* Liberibacter asiaticus’ chromosome. For example, *S. meliloti* is the nearest known relative of ‘*Ca.* Liberibacter asiaticus’, but when MOGs from ‘*Ca.* Liberibacter asiaticus’ were mapped on the *S. meliloti* chromosome, little overall synteny was observed. MOGs shared by ‘*Ca.* Liberibacter asiaticus’ and *S. meliloti* were dispersed all over their chromosomes (Fig. 1). Similar results were found when MOGs from ‘*Ca.* Liberibacter asiaticus’ were mapped on the circular chromosome of *A. tumefaciens* (Fig. S1). MOGs were distributed with relative uniformity among these genomes since the largest chromosomal regions void of MOGs shared by ‘*Ca.* Liberibacter asiaticus’ and *S. meliloti* and *A. tumefaciens* chromosomes was about 0.3 Mb in the larger genomes. When MOGs shared by *B. japonicum* and ‘*Ca.* Liberibacter asiaticus’ were mapped on respective linear chromosome representations, a similar lack of overall colinearity or macrosynteny of MOGs was observed. However, in contrast to the other genome comparisons, a region of 1.5 Mb in the *B. japonicum* chromosome failed to share any MOGs with ‘*Ca.* Liberibacter asiaticus’ (not shown). This region has genes that encode proteins required for the symbiotic interactions between *B. japonicum* and its plant hosts. The chromosomes of the free living bacteria are much larger than the ‘*Ca.* Liberibacter asiaticus’ chromosome, but *B. henselae* has a chromosome only slightly larger than that of ‘*Ca.* Liberibacter asiaticus’. As was observed with the larger genomes, when MOGs shared by ‘*Ca.* Liberibacter asiaticus’ and *B. henselae* were mapped, a similar lack of overall colinearity or macrosynteny of MOGs between the two linear genome representations was found, with a single region of the *B. henselae* genome devoid of MOGs shared with ‘*Ca.* Liberibacter asiaticus’ (Fig. S2).

Regions of microsynteny, colored according to functional class of the proteins in the COG database encoded by genes within the cluster, are illustrated (Fig. 1; Figs. S1, S2). For this purpose the functional classification from the COG database was used [23], [30]. For many of the clusters, the gene content by functional class was uniform. For example large clusters of genes related to cell surface, flagella, ribosomes and energy generation were conserved and homogenous. However, many of the MOGs, conserved within the Rhizobiales, contain genes of diverse functional classes, represented by black coloration (Fig. 1; Figs. S1, S2).

### Genes shared by ‘*Ca.* Liberibacter asiaticus’ with other Rhizobiales

**Figure 2:**
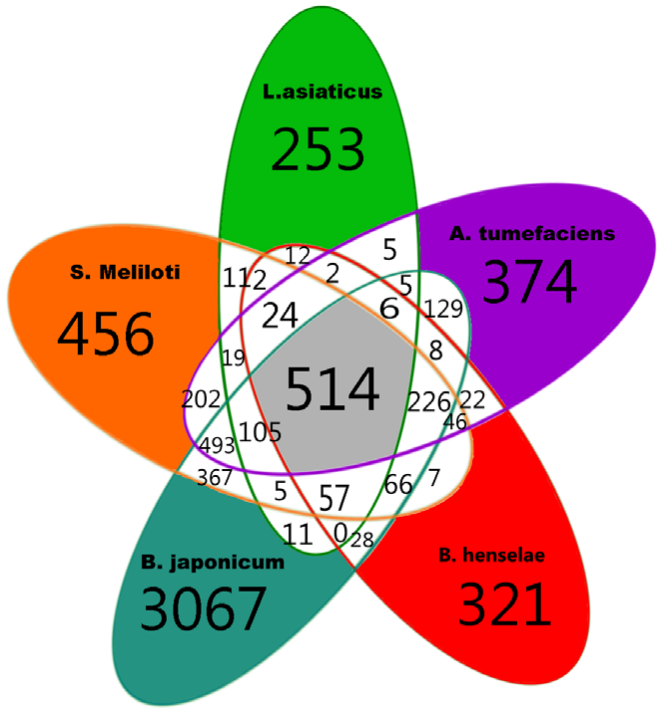
Venn diagram showing proteins either with or without orthologs shared among five diverse members of the Rhizobiales.

In addition to microsyntenous orthologous genes, or MOGs, the predicted protein products of all genes or ORFs (open reading frames) of the five species of the *Rhizobiales* studied were catalogued (Fig. 2). 514 orthologous genes are shared among all five species, and there were 226 additional orthologous genes found in four species but not found in ‘*Ca.* Liberibacter asiaticus’. There were an additional 57 genes shared by the chromosomes of *B. henselae*, *S. meliloti*, *B. japonicum* and ‘*Ca.* Liberibacter asiaticus’ but lacking from the *A. tumefaciens* circular chromosome. Interestingly, in a five-way Venn diagram comparison, only six orthologous genes not found in *S. meliloti* were shared by the four other species (Fig. 2). When the pathogens were considered separately, only two orthologous gene pairs were found in *B. henselae*, ‘*Ca.* Liberibacter asiaticus’ and *A. tumefaciens* but not in *S. meliloti* and *B. japonicum* and only five orthologous gene pairs shared exclusively by ‘*Ca.* Liberibacter asiaticus’ and *A. tumefaciens* (plant pathogens) (Table S2). Two of these had excellent e-values, a protein encoding a cation efflux pump (e^−64^) and a hypothetical protein (e^−37^). Twelve genes were shared only by *B. henselae* and ‘*Ca.* Liberibacter asiaticus’, both intracellular pathogens. Six of these had excellent e-values, including a DNA integrase/recombinase (2 e^−55^), a carboxynorspermidine decarboxylase (1 e^−122^). Both of these proteins interact with DNA. There was also a Na^+^/H^+^ antiporter (5 e^−48^), and a hypothetical protein with an excellent e-value (1 e^−149^). There were also five phage genes with less robust e-values shared between ‘*Ca.* Liberibacter asiaticus’ and *B. henselae* (Table S2).

Only eleven orthologous genes were shared exclusively by *S. meliloti* and ‘*Ca.* Liberibacter asiaticus’, the two nearest species phylogenetically among the five studied. Only one of the encoded proteins is fully annotated, a thiamine binding protein (5 e^−87^) with an apparent role in the uptake of thiamine from the host. The other 10 proteins shared exclusively by *S. meliloti* and ‘*Ca.* Liberibacter asiaticus’ are hypothetical proteins with relatively low homology scores so no functional insight can be gained from this comparison (Table S2).

### Proteins encoded by genes without orthologs between ‘Ca. Liberibacter asiaticus’ and other Rhizobiales

A total of 253 proteins are encoded by genes within ‘*Ca.* Liberibacter asiaticus’ but not shared with other bacteria included in this study (Fig. 2). Of this number most could not be annotated with known functions and are therefore hypothetical proteins. The remaining 63 proteins were assigned to COG categories [23], [30] with 12 genes encoding phage proteins. Of the remaining 51 proteins, 17 were classified in the information storage and processing category, 17 were classified in the cellular processes category and 17 were classified in the metabolic processes category (Table S3). Most of these proteins do not have any similar proteins in the other Rhizobiales studied, since only matches with positive e-values were found by P-Blast. In some cases homologs with relatively high e-values were found in one of the other species tested, but the annotations differed in the respective genomes, and so these are not considered to be matches (Table S3).

Among the proteins in the information storage and processing category and found only in ‘*Ca.* Liberibacter asiaticus’ are three proteins annotated as having SNF2 dead box helicase domains (ACT56602–ACT56603, ACT57668). There is also a protein GCN5- related N acetyltransferase domain (ACT57462). Proteins of this type interact with DNA-protein structures to unwind the DNA helix and are widespread in eukaryotic genomes where the interaction is with chromatin, but they have also been reported in prokaryotes [31]–[32]. The genes encoding these proteins are linked with genes encoding DNA ligase and proteins with VRR-NUC domains, which have nuclease activity. There is another group of genes (ACT57620–ACT57623) which include proteins with replicative helicase domains and a gene encoding a transcriptional regulator, suggesting roles for the products of these genes in transcription. The remaining proteins in this group include proteins with endo/exo nuclease and phosphatase domains. Taken together it appears that protein nucleic acid interactions in ‘*Ca.* Liberibacter asiaticus’ may differ in important details from those typical of the *Rhizobiales*.

There is also a group of 17 proteins found only in ‘*Ca.* Liberibacter asiaticus’ in the COG functional category of cellular processes. This includes a Type II DNA restriction and modification system encoded by ACT56990–ACT56987, a defensive function. There are also proteins involved in modifications of the cell surface. Encoded by ACT57391–ACT57394, a set of three proteins have domains indicating they may have roles in either adding sugars to the LPS layer or to an EPS layer of ‘*Ca.* Liberibacter asiaticus’. There is also a group of proteins annotated as part of cellular machinery responsible for assembly of pili. Besides serving as conduits for DNA transfer or type IV secretion, pili are responsible for cell-cell interactions either among bacteria making a biofilm [33]–[34] or in twitching motility [35], and so may be useful for ‘*Ca.* Liberibacter asiaticus’ in either the insect gut and salivary gland or in plant phloem cells. There is also a transporter protein for glucose unique to ‘*Ca.* Liberibacter asiaticus (ACT57316). This may be significant because of the sucrose rich environment in the plant phloem cells that the bacterium colonizes. In addition to proteins described in Table S3, ACT57581 encodes a protein with von Willebrand factor A domains. This protein is shared with *S. meliloti* and ‘*Ca.* Liberibacter solanacearum’, and such proteins in eukaryotes are found on the surface of blood cells. ACT57584 encodes a geranyl transtransferase shared with various *Bartonella* spp. and *Chlamydia pneumonia*. The presence of a glycosyl transferase and a preprotein translocase encoded nearby (ACT57587 and ACT57589) makes cell surface modification likely as their function.

Among the proteins in the COG functional category of metabolism and found only in ‘*Ca.* Liberibacter asiaticus’ is a set of seven proteins of the mevalonate pathway (ACT57489–ACT57495). In vertebrates this pathway leads to the synthesis of cholesterol, and therefore proteins encoded by this operon likely contribute to the cell membrane and may influence host/pathogen or host/vector interactions. There is also an ATP/ADP translocase (ACT56796) which likely functions in the uptake of ATP from the host cell [23]. ACT56857 encodes a serralysin not shared with other *Rhizobiales*. Serralysins are metallo proteases and in *Serratia marcesens* they modulate the mammalian inflammation response by specifically cleaving receptor proteins on the host cell surface [36].

There are also a number of phage-associated genes (Table S4). The phage genes encode a prophage antirepressor, a lysozyme, a terminase. The phage proteins can be annotated by Blast against Genbank, and domains can also be recognized with relatively strong matches to the Pfam database. One such domain is CRISPR in ACT56597 (4.79 e^−79^). The nucleic acid sequence of this protein has a series of palindromes and is thought to have a defensive function.

## Discussion

The early workers who created the proposed genus ‘*Candidatus* Liberibacter’ placed it within the α2-subdivision of the Proteobacteria based on sequence analysis of the 16S rRNA gene [11], [37]. Duan et al. created a phylogenetic tree of the α-Proteobacteria based on concatenated sequence alignments of 94 proteins. Their overall alignment of the α-Proteobacteria was the same as previously demonstrated [38] with the addition of ‘*Ca.* Liberibacter asiaticus’ firmly within the Rhizobiales as an “early branching member" of the Rhizobiaceae.

The significance of protein matches estimated by the e-statistic is subject to interpretation. Datasets for Microsyntenous Orthologous Genes (MOGs) were initially based on the conservative cut off criterion of a Blast-p e-value of 10^−10^ or less. Manual curation, with the annotations of proteins with higher e-values considered, identified additional probable homologs and increased MOG cluster size by about 10–15%. Many proteins were identified in ‘*Ca.* Liberibacter asiaticus’ but not shared with other members of the *Rhizobiales* tested. This determination was made when the value of the e-statistic between the protein from ‘*Ca.* Liberibacter asiaticus and proteins from the other members of the Rhizobiales tested was always positive, a decisive criterion.

Guerrero et al (2005) found conserved clusters of orthologous genes that are shared among free-living Rhizobiales, including *Mesorhizobium loti* and *Brucella melitensis*, and mapped them with an approach similar to ours [4]. Substantial overall macro synteny in the arrangement of blocks of orthologous genes was observed. In that study the genomes compared were less phylogenetically distinct and more similar in size. Our identification of MOGs between ‘*Ca.* Liberibacter asiaticus’ and other *Rhizobiales* differs from theirs since we observed little overall macrosynteny in the circular chromosomes of ‘*Ca.* Liberibacter asiaticus’ and other members of the *Rhizobiaceae, S. meliloti and A. tumefaciens*. Extensive genomic rearrangement accompanied the severe reduction in chromosome size as ‘*Ca.* Liberibacter asiaticus’ adapted to specialized intracellular niches. In spite of the extensive genomic rearrangements we did find at least 50 blocks of microsyntenous orthologous genes containing 283 of the 514 shared conserved orthologous genes. These MOGs were found in all three families of the *Rhizobiales* studied (*Rhizobiaceae*, *Bartonellaceae* and *Bradyrhizobiaceae*) and contained an average of about six genes but contained as few as three or as many as 31 genes. This conserved microsynteny of core genes helps define the α-Proteobacterial order Rhizobiales.

*B. henselae* lives inside erythrocytes of cats and has undergone an extensive genomic reduction similar to ‘*Ca.* Liberibacter asiaticus’. Such genomic reductions are thought to be part of a convergent evolutionary process [39], which leads to genomes with low mol % GC content [40]. As with the other chromosomes compared, *B. henselae* and ‘*Ca.* Liberibacter asiaticus’ lack overall chromosomal macrosynteny, consistent with the different evolutionary histories leading to intracellular parasitism of citrus/psyllids versus cats/fleas. Nonetheless, the two intracellular microorganisms do share 50 sets of MOGs with the free-living members of the *Rhizobiales* and with each other. The reduction of genome size in diverse obligate intracellular parasites such as ‘*Ca.* Liberibacter asiaticus’ leads to the retention of a core of 100 genes encoding the most essential protein COGs [39]. The ‘*Ca.* Liberibacter asiaticus’ genome has genes that encode all 100 of these essential protein COGs, although the genes encoding the DNA polymerase III alpha and gamma/tau subunits have characteristics of pseudogenes [23]. Our bioinformatic analyses suggest that the alpha subunit gene is likely to be functional and is, in any case the only option available for DNA replication in ‘*Ca.* Liberibacter asiaticus’. The gamma tau subunit may also be functional but analysis of gene sequence data is not compelling [40].

We observed two types of MOGs conserved in the *Rhizobiales*. The first type is composed of genes from a common pathway or structure. The best example of this type of MOG was the large cluster of ribosomal RNA and ribosomal protein-encoding genes (ACT 56739–ACT56709). These 31 genes are completely conserved within MOGs shared in all five bacteria compared. Other examples of this type of microsyntenous block, present in all five members of the *Rhizobiales* included in this study, include a cluster of four genes encoding four subunits of a cytochrome-O-ubiquinol oxidase assembly (ACT56945–ACT56947), a cluster of cell wall synthesis genes (ACT57578–ACT57568) and a cluster of genes related to sulfur metabolism (ACT57532–57536) (Table S1). Clusters of flagellar genes in ‘*Ca.* Liberibacter asiaticus’ are orthologous with flagellar operons in *S. meliloti*, *B. japonicum* and *A. tumefaciens*, but flagellar genes were not found in *B. henselae*. The second type of block of microsyntenous orthologous genes was composed of genes without an obvious common pathway or function. An example of this type include a block of 3 genes identified as encoding an arginyl-tRNA synthetase, a deoxyguanosine triphosphate triphosphohydrolase-like protein and a HesB iron-sulfur cluster assembly protein (ACT56758–ACT56760). Another example of a diverse but conserved MOG is found at ACT56879–ACT56876 encoding a large ribosomal subunit pseudouridine synthase B, a conserved hypothetical protein, a myo-inositol monophosphate family protein, and another conserved hypothetical protein. We also found a conserved gene presumably involved in LPS biosynthesis linked to genes encoding a conserved hypothetical protein and a DNA mismatch repair protein (ACT56874–56872). The microsyntenous blocks we observe as common to all five members of the Rhizobiales representing three families studied are remarkably conserved (Table S1) despite extensive genomic rearrangements (Fig. 1; Figs S1, S2), concurrent with dramatic reduction or expansion in genome size and both niche divergence and specialization. There are different explanations proposed for the conservation of gene order during evolution, including efficiency of co-regulation and a selfish gene model [4], [41]. Computer simulations have been used to show that such clusters are not inevitable but are influenced by population size, the number of genes in a pathway and horizontal gene transfer [41].

Members of the *Rhizobiales* have evolved from a common ancestor to occupy several diverse niches. Fifty clusters of conserved genes are found in all five taxons, in spite of extreme physiological and genomic differences. We also found a large number of orthologous genes not in MOGs but that are shared among all of the species. MOGs and other conserved orthologous genes encode proteins that carry out core metabolic functions. Other MOGs and conserved orthologous genes are restricted to subsets of related species and seem likely to encode proteins whose functions are related to niche-specific genetic and physiological adaptations.

A number of the 253 proteins encoded by genes found only in ‘*Ca.* Liberibacter asiaticus’ have roles in nucleic acid interactions. Among these, there is a large set of genes with functions ascribed to DNA replication or transcription. The genome of ‘*Ca.* Liberibacter asiaticus’ is highly reduced and very low in mol% G+C [23], [40]. There are several unshared proteins with DNA helicase domains, and a set of four genes with functions protecting or binding to DNA. These proteins as well as proteins with exo/endonuclease and phophatase domains, which share an affinity for DNA, suggest that DNA-protein interactions require a large suite of proteins not found in other *Rhizobiales*. This set of proteins unique to ‘*Ca.* Liberibacter asiaticus’ in the *Rhizobiales* tested is bolstered by two proteins shared with *B. henselae*, an integrase recombinase and the carboxynorspermidine decarboxylase (Table S2). The low mol% G+C genome shared by these organisms is thought to conserve ATP [42], since DNA unwinding for either transcription or replication must be done at the expense of ATP [43]. It is likely that the proteins that interact with DNA and are unique to ‘*Ca.* Liberibacter asiaticus’ have coevolved with the low mol% G+C composition of the genome. The proteins with SNF2 helicase and acetyltransferase domains encoded by the ‘*Ca.* Liberibacter asiaticus’ genome may also directly mediate host pathogen interactions. Bacterial pathogens often express proteins with these domains that participate in the remodeling of the host chromatin leading to altered patterns of gene expression by the host in response to infection [44].

The proteins encoded by ‘*Ca.* Liberibacter asiaticus’ but not shared with other members of the *Rhizobiales* are also of great interest because they may contribute to interactions specific to the pathogen, its host and its vector. Some may condition host cells for intracellular colonization or for insect vector-specific colonization and transmission. In particular, the surface of ‘*Ca.* Liberibacter asiaticus’ appears to be functionally different from that of the other members of the *Rhizobiales* tested. There are seven genes that comprise a pathway to synthesize cholesterol not present in the other Rhizobiales tested. Proteins that modify the cell surface include enzymes that add sugar moieties to LPS or EPS, as well as proteins belonging to the large family of von Willebrand factor Type A domains. Proteins in this family typically are exposed on the cell surface and participate in large supramolecular structures. Therefore these proteins as well as the probable pilus and the operons encoding the cholesterol biosynthetic pathway could help permit compatible interaction of ‘*Ca.* Liberibacter asiaticus’ and its plant and insect hosts.

Twelve proteins shared only with *B. henselae*, another intracellular pathogen with an insect vector, included 4 hypothetical proteins and 5 phage related proteins. Bacteriophage have been observed in lytic attacks on ‘*Ca.* Liberibacter asiaticus’ in infected phloem cells of citrus [45]. Bacteriophage may also act to modify the host genome, and may play a role in the genomic reduction experienced by both *B. henselae* and ‘*Ca.* Liberibacter asiaticus’. Thus it is interesting that bacteriophage genes are shared by *B. henselae* and ‘*Ca.* Liberibacter asiaticus’. There is also a protein that likely functions as a Na+/H+ antiporter (5.00 e^−48^). This is important for the maintenance of intracellular homeostasis. Another set of five proteins shared between ‘*Ca.* Liberibacter asiaticus’ and *A. tumefaciens* may also be used to control other aspects of host/pathogen interaction, although the lack of more uniquely common genes between these two plant pathogens is consistent with their use of widely divergent mechanisms to colonize plants and induce disease. One of the proteins ‘*Ca.* Liberibacter asiaticus’ (ACT56967) with a homolog encoded by *A. tumefaciens* (1.00 e-64) is likely to be a component of a cation efflux system. Another cation efflux protein is uniquely shared between the *S. meliloti* megaplasmid pSymA and ‘*Ca.* Liberibacter asiaticus’, and is likely to play a significant role in maintenance of cellular homeostasis [46].

We have compared the total genome of ‘*Ca.* Liberibacter asiaticus,’ comprised of a single circular chromosome, against the circular chromosomes of other members of the *Rhizobiales*. We have defined clusters of microsyntenous observed genes (MOGs) shared by the entire set of bacteria studied. This set of genes, engaged in core metabolic functions, contribute to the definition of the order *Rhizobiales*. We have also identified sets of genes unique to the plant pathogen ‘*Ca.* Liberibacter asiaticus’, and which presumably are important to its interactions with both plant hosts and insect vectors. Among this group of proteins we found a large set of proteins engaged in DNA or RNA interactions, including proteins that may modify the expression of host genes, function as cation efflux and Na+/H+ antiporters to maintain intracellular homeostasis, participate in cell surface modifications and in the production of lipids for the cell membrane. The extra chromosomal elements of *S. meliloti* or *A. tumefaciens* were not included in this study. Extra chromosomal genetic elements often encode factors important to the host/pathogen interactions [47]. We have found several interesting regions conserved between the *S. meliloti* pSymA and the ‘*Ca.* Liberibacter asiaticus’ genome, and these are described separately [46].

## Materials and Methods

### Identification of regions of microsynteny between ‘*Ca.* Liberibacter asiaticus’ and members of the Rhizobiales

The circular chromosomes of four members of the *Rhizobiales* were selected for comparative chromosome analysis with ‘*Ca.* Liberibacter asiaticus’. To identify orthologous proteins, predicted amino acid sequences (‘Ca. Liberibacter asiaticus’ strain psy62, CP001677.1; *Bartonella henselae* strain Houston-1, BX897699; *Sinorhizobium meliloti* strain 1021, AL591688; *Bradyrhizobium japonicum* strain USDA 110, BA000040 and *Agrobacterium tumefaciens* strain C58, AE007869) were downloaded from NCBI. Extra chromosomal DNA sequences were not included in this study. Using default BLAST parameters, each predicted amino acid sequence from the ORFs identified on a chromosome of this group of strains was BLASTed against the predicted amino acid sequences of the ORFs on the other chromosomes of this group of phyogenetically related strains. Perl scripts and Excel spreadsheets were created to identify hits between genomes with low, negative e-values. Microsyntenous regions conserved between ‘*Ca.* Liberibacter asiaticus’ and the circular chromosomes of four other members of the Rhizobiales were identified by using the following criteria: (1) a minimum of three orthologous genes in succession, (2) in an identical order and (3) with predicted protein products that shared Blast alignment e-values of less than 10^−10^. After review and manual curation by the authors, some blocks were extended based on likely gene co-regulation, if for example, orthologous genes with low e-values were separated from other genes from the same pathway or structure by one or two genes with e-values that did not meet the e^−10^ threshold. The choice of the e-value to use for any purpose involves compromise. The use of the e-value threshold of 1 e^−10^ as the starting point for defining the MOG groups was relatively conservative, but particularly when supplemented by manual curation, allowed for the inclusion of more genes into MOGs than would a more conservative criterion. The resulting MOGs were defined with a bias to include only genes with solid matches.

### Mapping microsyntenous regions

Regions of microsyntenous orthologous genes or MOGs among or between ‘*Ca.* Liberibacter asiaticus’ and other members of the *Rhizobiales* were defined as described above. The names of the orthologous genes, the functions of the proteins and the beginning and ending codons of the corresponding genes were entered in a spreadsheet ordered with reference to the ‘*Ca.* Liberibacter asiaticus’ genome. Microsyntenous regions were mapped onto linear representations of their respective genomes using Corel graphics software with vector graphic technology and were colored according to the COG functional categories of the proteins encoded [23], [30].

### Proteins encoded by genes unique to ‘*Ca.* Liberibacter asiaticus’ or shared with other Rhizobiales studied

Blast-p was used to compare the proteomes encoded by each of the five circular chromosomes with the other four. A 5-way Venn diagram was created summarizing orthologous genes [e^−10^ or less] shared among all Rhizobiales. Manual curation of the datasets allowed protein pairs with slightly higher e-values to be recognized as orthologous based on genomic contexts, so that orthologous proteins, if present, would be identified. However, proteins from ‘*Ca.* Liberibacter asiaticus’ were determined to be absent in the other members of the Rhizobiales tested only if all proteins matched in the other species tested with BlastP produced positive e-values. This decisive criterion removes any ambiguity from our identifications of proteins that are unique to ‘Ca. Liberibacter asiaticus’ among the members of the *Rhizobiales* tested. All proteins of ‘*Ca.* Liberibacter asiaticus’ encoded by genes not shared with the other *Rhizobiales* were subjected to BLASTP against Genbank and the CDD database in order to find possible orthologs in other organisms. SMART analysis of each amino acid sequence was also used to find domains [48]. Proteins with annotations were categorized according to the COG database [30]. Hypothetical proteins were further analyzed by chromosomal position and length of protein to provide further insight into possible functionalities.

## Supporting Information

Figure S1 **Comparative organization of the chromosomes of ‘** ***Ca.*** **Liberibacter asiaticus’ and** ***A. tumefaciens*** **.** Microsyntenous orthologous genes (MOGs) were identified and plotted on respective chromosomes. Lines connect identical blocks of genes. The upper chromosome is that of ‘*Ca.* Liberibacter asiaticus’, 1.2 Mb. The bottom chromosome is that of chromosome of *A. tumefaciens* (2.8 Mb).
(TIF)

Figure S2 **Comparative organization of the circular chromosomes of ‘** ***Ca.*** **Liberibacter asiaticus’ and** ***B. henselae*** **.** Microsyntenous orthologous genes (MOGs) were identified and plotted on respective chromosomes. Lines connect identical blocks of genes. The upper chromosome is that of ‘*Ca.* Liberibacter asiaticus’, 1.2 Mb. The bottom chromosome is that of chromosome of *B. henselae* (1.9 Mb).
(TIF)

Table S1 **Proteins encoded by microsyntenous orthologous genes (MOGs) in diverse members of the** ***Rhizobiales*** **.** These proteins are encoded by the circular chromosomes of ‘*Ca.* Liberibacter asiaticus, *S. meliloti*, *A. tumefaciens*, *B. japonicum* and *B. henselae* with e-values of −10 or lower and in groups of 3 or more, and may be thought of as defining the order, in a sense.
(DOCX)

Table S2 **Proteins annotated from the ‘** ***Ca.*** **Liberibacter asiaticus circular chromosome and shared with only one other member of the Rhizobiales studied** ***B. henselae*** **,** ***S. meliloti*** **,** ***A. tumefaciens*** **,** ***B. japonicum*** **.**
(DOCX)

Table S3 **Proteins encoded by the ‘** ***Ca.*** **Liberibacter asiaticus chromosome that did not have orthologs in** ***S. meliloti*** **,** ***A. tumefaciens*** **,** ***B. japonicum*** **and** ***B. henselae*** **.**
(DOCX)

Table S4 **Proteins encoded by the ‘** ***Ca.*** **Liberibacter asiaticus’ chromosome that did not have orthologs in** ***S. meliloti*** **,** ***A tumefaciens*** **,** ***B. japonicum*** **and** ***B. henselae*** **and which encode phage related genes.**
(DOCX)
