# Supplementary figures and images for: Conservation of Gene Order and Content in the Circular Chromosomes of ‘Candidatus Liberibacter asiaticus’ and Other Rhizobiales
Source: PLoS One. 2012 Apr 4;7(4):e34673. doi: 10.1371/journal.pone.0034673 (PMC3319617; doi:10.1371/journal.pone.0034673)

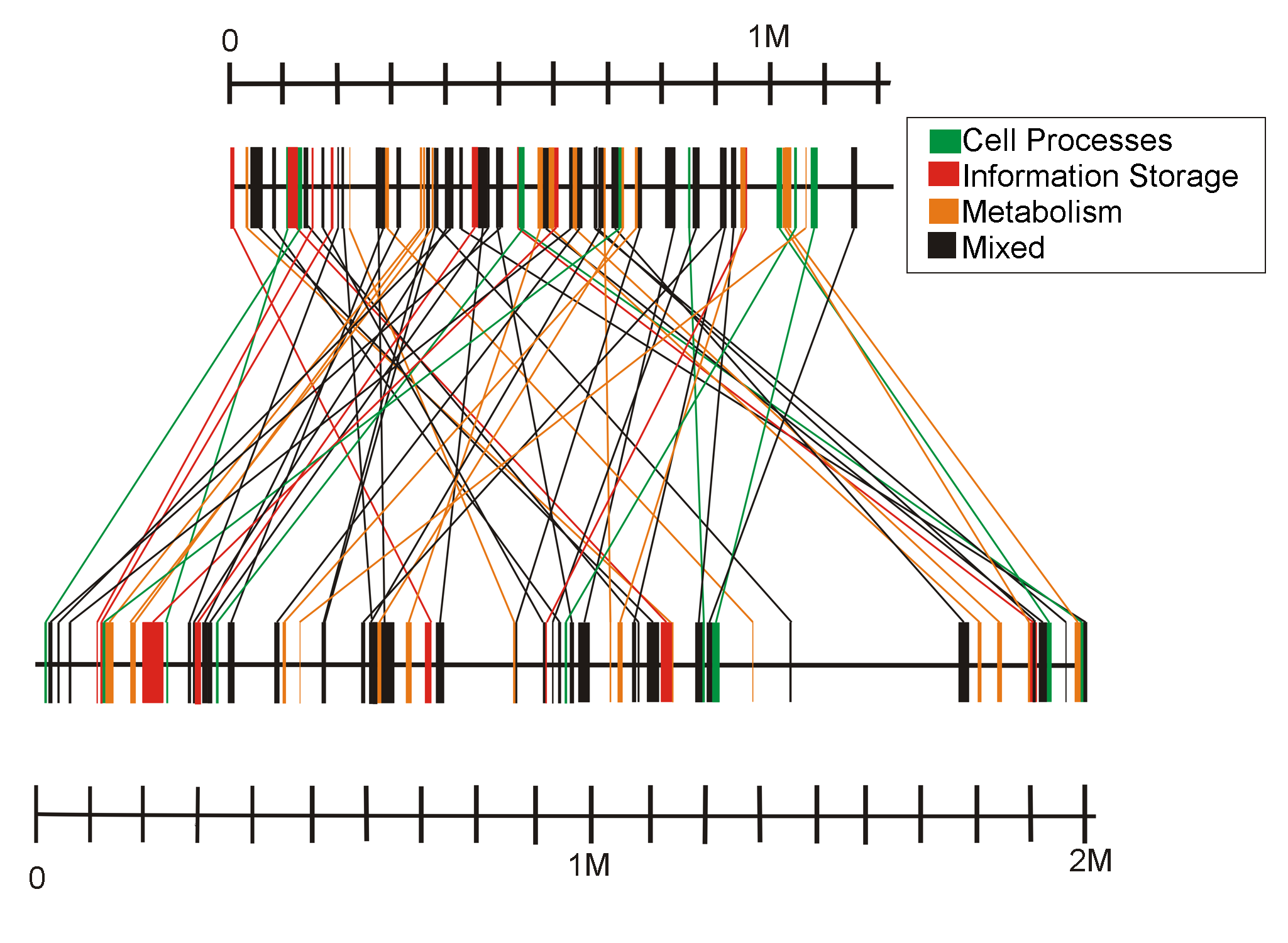

Supplement: Figure S2 — Comparative organization of the circular chromosomes of ‘ Ca. Liberibacter asiaticus’ and B. henselae . Microsyntenous orthologous genes (MOGs) were identified and plotted on respective chromosomes. Lines connect identical blocks of genes. The upper chromosome is that of ‘Ca. Liberibacter asiaticus’, 1.2 Mb. The bottom chromosome is that of chromosome of B. henselae (1.9 Mb). (TIF) [file pone.0034673.s002.tif]
