# Supplementary material for: Conservation of Gene Order and Content in the Circular Chromosomes of ‘Candidatus Liberibacter asiaticus’ and Other Rhizobiales
Source: PLoS One. 2012 Apr 4;7(4):e34673. doi: 10.1371/journal.pone.0034673 (PMC3319617; doi:10.1371/journal.pone.0034673)
Supplement: Table S1 — Proteins encoded by microsyntenous orthologous genes (MOGs) in diverse members of the Rhizobiales . These proteins are encoded by the circular chromosomes of ‘Ca. Liberibacter asiaticus, S. meliloti, A. tumefaciens, B. japonicum and B. henselae with e-values of −10 or lower and in groups of 3 or more, and may be thought of as defining the order, in a sense. (DOCX) [file pone.0034673.s003.docx]

**Table S1.** Proteins encoded by microsyntenous orthologous genes (MOGs) in diverse members of the *Rhizobiales*. These proteins are encoded by the circular chromosomes of ‘*Ca*. Liberibacter asiaticus, *S. meliloti*, *A. tumefaciens*, *B. japonicum* and *B. henselae* with e-values of -10 or lower and in groups of 3 or more, and may be thought of in a sense as defining the order.

| **Protein ID ^a^** | **FCC ^b^** | **COG ^c^** | **Description** |
| --- | --- | --- | --- |
| ACT56621 | K | COG0250 | NusG transcription anti-terminator protein |
| ACT56620 | J | COG0080 | RplK L11 50S ribosomal protein |
| ACT56619 | J | COG0081 | RplA L1 50S ribosomal protein |
| ACT56618 | J | COG0244 | RplJ L10 50S ribosomal protein |
| ACT56617 | J | COG0222 | RplL 50S ribosomal protein L7/L12 |
| ACT56616 | K | COG0085 | DNA-directed RNA polymerase beta chain 140 kd |
| ACT56615 | K | COG0086 | DNA-directed RNA polymerase beta chain 160 kd |
| ACT56615 | K | COG0250 | NusG transcription anti-terminator protein |
|  |  |  |  |
| ACT56646 | E | COG1126 | L-amino acid ABC transporter assembly ATPase polar amino acid transporter |
| ACT56645 | E | COG0765 | L-amino acid ABC transporter assembly amino acid transporter protein |
| ACT56644 | E | COG4597 | L-amino acid ABC transporter assembly L-amino acid permease |
| ACT56643 | E | COG0834 | L-amino acid ABC transporter assembly membrane-spanning periplasmic transporter |
|  |  |  |  |
| ACT56657 | L | COG0178 | excinuclease ABC subunit A |
| ACT56656 | L | COG0629 | single-strand DNA-binding (ssb) protein |
| ACT56655 | L | COG0188 | DNA gyrase subunit A |
| ACT56654 | H | COG0669 | phosphopantetheine adenylyltransferase |
|  |  |  |  |
| ACT56691 | F | COG0125 | thymidylate (dTMP) kinase |
| ACT56690 | L | COG0470 | DNA polymerase III/ ATPase delta subunit |
| ACT56689 | J | COG0143 | probable methionyl-tRNA synthetase |
| ACT56688 | L | COG0084 | TatD family deoxyribonuclease |
| ACT56687 | V | COG1235 | metal-dependent hydrolase or betalactamase B superfamily protein |
|  |  |  |  |
| ACT56739 | J | COG0048 | S12 30S ribosomal protein |
| ACT56738 | J | COG0049 | S7 30S ribosomal protein |
| ACT56737 | J | COG0480 | GTPase elongation factor G |
| ACT56736 | J | COG0050 | GTPase elongation factor Tu |
| ACT56735 | J | COG0051 | S10 ribosomal protein |
| ACT56734 | J | COG0087 | L3 50S ribosomal protein |
| ACT56733 | J | COG0088 | L4 50S ribosomal protein |
| ACT56732 | J | COG0089 | L23 50S ribosomal protein |
| ACT56731 | J | COG0090 | L2 50S ribosomal protein |
| ACT56730 | J | COG0185 | S19 ssu ribosomal protein |
| ACT56729 | J | COG0091 | L22 50S ribosomal protein |
| ACT56728 | J | COG0092 | S3 30S ribosomal protein |
| ACT56727 | J | COG0197 | 50S ribosomal protein L16 |
| ACT56726 | J | COG0255 | 50S ribosomal protein L29 |
| ACT56725 | J | COG0186 | S17 30S ribosomal protein |
| ACT56724 | J | COG0093 | L14 50S ribosomal protein |
| ACT56723 | J | COG0198 | L24 50S ribosomal protein |
| ACT56722 | J | COG0094 | L5 50S ribosomal protein |
| ACT56721 | J | COG0199 | S14 30S ribosomal protein |
| ACT56720 | J | COG0096 | S8 30S ribosomal protein |
| ACT56719 | J | COG0097 | L6 50S ribosomal protein |
| ACT56718 | J | COG0256 | L18 50S ribosomal protein |
| ACT56717 | J | COG0098 | S5 30S ribosomal protein |
| ACT56716 | J | COG1841 | L30 50S ribosomal protein |
| ACT56715 | J | COG0200 | L15 50S ribosomal protein |
| ACT56714 | J | COG0201 | secY subunit of preprotein translocase |
| ACT56713 | J | COG0563 | adenylate kinase |
| ACT56712 | J | COG0099 | RpsM L13 30S ribosomal protein |
| ACT56711 | J | COG0100 | L11 30S ribosomal protein |
| ACT56710 | J | COG0202 | DNA-directed RNA polymerase subunit alpha |
| ACT56709 | J | COG0203 | RplQ L17 50S ribosomal protein |
|  |  |  |  |
| ACT56707 | E | COG0137 | argininosuccinate synthase (not in B. henselae) |
| ACT56706 | T | COG1217 | membrane GTPase /tyrosine-phosphorylated |
| ACT56705 | M | COG 0762 | integral membrane protein |
|  |  |  |  |
| ACT56743 | U | COG0466 | ATP-dependent protease Lon type |
| ACT56744 | U | COG1219 | ATP-dependent protease ATP-binding subunit ClpX |
| ACT56745 | U | COG0740 | proteolytic subunit of ATP-dependent ClpP protease |
|  |  |  |  |
| ACT56758 | J | COG0018 | arginyl-tRNA synthetase |
| ACT56759 | J | COG0232 | deoxyguanosinetriphosphate triphosphohydrolyase-like protein |
| ACT56760 | P | COG0316 | HesB iron-sulfur cluster assembly accessory protein |
|  |  |  |  |
| ACT56768 | J | COG0101 | tRNA pseudouridine synthetase |
| ACT56769 | J | COG0223 | methionyl-tRNA formyltransferase |
| ACT56770 | J | COG0242 | N-formylmethionly-tRNA deformylase |
|  |  |  |  |
| ACT56772 | I | COG1960 | acyl-CoA dehydrogenase protein |
| ACT56773 | E | COG0540 | aspartate carbomyltransferase catalytic subunit |
| ACT56774 | E | COG0044 | dihydroorotase |
| ACT56775 | I | COG0344 | membrane predicted glycerol-3-phosphate acyltransferase |
|  |  |  |  |
| ACT56789 | J | COG0072 | phenylalanyl-tRNA synthetase subunit beta |
| ACT56790 | J | COG0564 | pseudouridylate synthase 23S RNA-specific |
| ACT56791 | J | COG0292 | Ribosomal subunit L20 |
| ACT56792 | J | COG0291 | Ribosomal subunit L35 |
|  |  |  |  |
| ACT56810 | R | COG1028 | dehydrogenase of short chain alcohols |
| ACT56809 | F | COG0034 | glutamine phosphoribosylpyrophosphate amidotransferase |
| ACT56808 | V | COG1286 | uncharacterized membrane protein required for colicin V production protein |
| ACT56807 | O | COG1066 | ATP-dependent serine protease |
| ACT56806 | E | COG0787 | alanine racemase |
| ACT56805 | L | COG0305 | replicative DNA helicase |
| ACT56804 | J | COG0359 | L9 ribosomal protein |
| ACT56803 | J | COG0238 | S18 ribosomal protein |
| ACT56802 | J | COG0360 | S6 30S ribosomal protein |
|  |  |  |  |
| ACT56822 | E | COG5682 | glycine/serine hydroxymethyltransferase (SHMT) |
| ACT56821 | H | COG1885 | ribD riboflavin synthesis E.C. 2.1.2.1 ?? |
| ACT56820 | H | COG0307 | alpha chain of riboflavin synthase |
| ACT56819 | H | COG0054 | beta chain of riboflavin synthase |
| ACT56818 | K | COG0781 | NusB transcription antitermination protein B |
|  |  |  |  |
| ACT56935 | I | COG0623 | enoyl-ACP-[acyl carrier protein] reductase |
| ACT56934 | I | COG0454 | acetyltransferase or 3-oxoacyl-ACP synthetase I |
| ACT56933 | I | COG0764 | 3-hydroxymyristoyl/3-hydroxydecanoyl-(acyl carrier protein dehydratase |
| ACT56932 | S | COG4530 | Hypothetical protein |
|  |  |  |  |
| ACT56871 | J | COG0030 | dimethyladenosine transferase (rRNA methylation) |
| ACT56870 | E | COG1995 | pyridoxyl phosphate biosynthesis protein |
| ACT56869 | U | COG0760 | peptidyl-prolyl isomerase parvulin-like protein |
| ACT56868 | M | COG1452 | OstA organic solvent tolerance protein |
| ACT56867 | R | COG0795 | Predicted transmembrane permease protein |
| ACT56866 | R | COG0795 | predicted permease protein |
| ACT56865 | U | COG0260 | leucyl aminopeptidase (not in Bartonella henselae) |
| ACT56864 | L | COG2927 | DNA polymerase III chi |
| ACT56863 | F | COG0105 | nucleoside diphosphate kinase |
|  |  |  |  |
|  |  |  |  |
| ACT56879 | J | COG1187 | 16S r-RNA uridine-516 pseudouridylate synthase |
| ACT56878 | F | COG0742 | N6-adenine-specific methylase |
| ACT56877 | I | COG0483 | myo-inositol monophosphatase family protein |
| ACT56876 | S |  | conserved hypothetical protein |
| ACT56875 | I | COG1519 | 3-deoxy-D-manno-octulosonic-acid transferase |
| ACT56874 | M,I | COG1663 | tetra-acyldisaccharide-1-P 4’-kinase |
| ACT56873 | S | COG3908 | uncharacterized conserved protein |
| ACT56872 | L | COG0323 | mutL DNA mismatch repair ATPase protein |
|  |  |  |  |
| ACT56882 | F | COG0462 | phosphoribosyl pyrophosphate synthase, i.e., a ribose phosphate pyrophospho kinase |
| ACT56883 | S | COG1565 | conserved uncharacterized hypothetical protein |
| ACT56884 | I | COG0682 | prelipoprotein diacylglyceryl transferase |
|  |  |  |  |
| ACT56945 | C | COG1845 | heme/copper type cytochrome o quinol oxidase, subunit 3 |
| ACT56946 | C | COG0843 | heme/copper type cytochrome quinol |
| ACT56947 | C | COG1622 | heme/copper type cytochrome quinol |
|  |  |  |  |
| ACT56950 | M | COG1651 | protein DSBA double sulfide bond-forming enzyme |
| ACT56951 | S | COG5389 | conserved bacterial protein |
| ACT56952 | L | COG1194 | MutY A/G-specific adenine glycosylase |
|  |  |  |  |
| ACT57182 | D | COG0552 | signal recognition particle GTPase |
| ACT57183 | M | COG0253 | diaminopimelate epimerase |
| ACT57184 | T | COG05641 | signal recognition particle protein |
| ACT57185 | J | COG0228 | S16 30S ribosomal protein |
| ACT57186 | J | COG0806 | RimM protein required for 16S rRNA processing protein |
| ACT57187 | J | COG0336 | tRNA(guanine-N(1)-)-methyltransferase |
| ACT57188 | J | COG0336 | ribosomal protein L19 |
|  |  |  |  |
| ACT57300 | H | COG1072 | Pantothenate kinase |
| ACT57301 | O | COG5405 | ATP dependent protease ClpYQ |
| ACT57302 | O | COG1220 | ATP-dependent protease ATP-binding subunit |
|  |  |  |  |
| ACT57298 | T | COG2202 | hybrid sensor histidine kinase with a FOG PAS/PAC domain |
| ACT57297 | L | COG0802 | ATPase or kinase (AddB double strand break repair protein) |
| ACT57296 | L | COG3893 | inactivated superfamily 1 helicase |
|  |  |  |  |
| ACT57283 | K | COG1158 | Rho transcription termination factor |
| ACT57282 | J | COG0486 | tRNA modification GTPase |
| ACT57281 | D | COG0357 | cell division and chromosome partitioning protein |
| ACT57280 | D | COG0445 | cell division and chromosome partitioning protein |
| ACT57279 | D | COG1192 | cell division and chromosome partitioning protein |
| ACT57278 | K | COG1475 | transcriptional regulator |
|  |  |  |  |
| ACT57273 | J | COG0495 | leucyl tRNA synthtase |
| ACT57274 | S | COG0325 | enzyme with a TIM-barrel fold |
| ACT57275 | J | COG0495 | Leucyl-tRNA synthetase |
|  |  |  |  |
| ACT56897 | K | COG1393 | predicted transcriptional regulator |
| ACT56898 | I | COG0815 | apolipoprotein N-acyltransferase |
| ACT56899 | U | COG1293 | hemolysin and related proteins with CBS domains |
| ACT56900 | R | COG0319 | predicted metal-dependent hydrolase |
| ACT56901 | K | COG0621 | 2-methylthioadenine synthase |
| ACT56902 | U,O | COG1214 | putative molecular chaperone , inactive homolog of metal-dependent hydrolyases |
|  |  |  |  |
| ACT57252 | J | COG0052 | S2 30S ribosomal protein |
| ACT57253 | L | COG0537 | HIT histidine triad cell cycle protein |
| ACT57254 | E | COG2171 | 2,3,4,5-tetrahydropyridine-2,6-carboxylate N-succinyltransferase |
| ACT57255 | M | COG0624 | succinyl-diaminopimelatesuccccinyelate desuccinylase |
| ACT57256 | K | COG4957 | RosR/MucR-like transcription regulator protein |
| ACT57257 | J,K | COG1185 | polynucleotide phosphorylase/polyadenylase |
| ACT57258 | J | COG0184 | Ribosomal protein S15P/S13E |
|  |  |  |  |
| ACT57242 | M | COG1043 | ACP [acyl carrier protein]-UDP-N-acetylglucosamine O-acyltransferase |
| ACT57243 | M,I | COG0764 | 3-hydroxymyristoyl/3-hydroxydecanoyl–(ACP) dehydratase |
| ACT57244 | M,I | COG1044 | UDP-3-O-[3-hydroxy-myristoyl]glucosamine N-acyltransferase |
| ACT57245 | M | COG4775 | OMA87 surface antigen/ protective outer membrane protein |
| ACT57246 | M | COG0750 | membrane-associated Zn-dependent protease |
| ACT57247 | M,I | COG0575 | CDP-diglyceride synthase |
|  |  |  |  |
| ACT57224 | L | COG1530 | ribonuclease G and E |
| ACT57225 | M | COG5009 | Membrane carboxypeptidase / penicillin binding protein |
| ACT57226 | J | COG1186 | peptide chain release factor 2 |
| ACT57227 | R | COG 0061 | sugar kinase |
|  |  |  |  |
| ACT57119 | J | COG0261 | 50S ribosomal protein L21 |
| ACT57120 | J | COG0211 |  |
| ACT57121 | J | COG0536 | GTP(ase) protein |
| ACT57122 | H | COG1057 | nicotinate-nucleotide adenylyl-transferase |
|  |  |  |  |
| ACT57176 | P | COG0704 | phosphate uptake regulator |
| ACT57177 | P | COG1117 | conserved tetra-polypeptide ABC assembly for an ATPase phosphate transporter |
| ACT57178 | P | COG0581 | conserved tetra-polypeptide ABC assembly for an ATPase phosphate transporter |
| ACT57179 | P | COG0573 | conserved tetra-polypeptide ABC assembly for an ATPase phosphate transporter |
| ACT57180 | P | COG0226 | conserved tetra-polypeptide ABC assembly for an ATPase phosphate transporter |
|  |  |  |  |
|  |  |  |  |
| ACT57142 | I,M | COG28772 | 3-deoxy-D-manno-octulosonic acid (KDO) 8-phosphate synthetase |
| ACT57143 | R | COG0148 | enolase |
| ACT57144 | D | COG2919 | septum formation/ cell division initiator |
| ACT57145 | I | COG1071 | E1 component dehydrogenase alpha |
| ACT57146 | I | COG0022 | pyruvate dehydrogenase component beta [transketolase central] |
| ACT57147 | I | COG0508 | pyruvate dehydrogenase complex dihydrolipoamide dehydogense |
| ACT57148 | I | COG1249 | lipoyl synthetase not in Bradyrhizobium |
| ACT57149 | I | COG0320 | lipoyl synthase |
|  |  |  |  |
| ACT57096 | C | COG0661 | 2-polyprenylphenol 6-hydroxylase ubiquinone biosynthesis protein |
| ACT57097 | C | COG2226 | ubiquinone/ menaquinone biosynthesis methyltransferase protein |
| ACT57098 | L | COG0266 | formamidopyrimidine-DNA glycosylase |
|  |  |  |  |
| ACT57092 | L | COG1198 | primosomal protein N |
| ACT57091 | C | COG0712 | subunit delta of F0F1-type ATP synthetase |
| ACT57090 | C | COG0056 | subunit alpha of F0F1-type ATP synthetase |
| ACT57089 | C | COG0224 | subunit gamma of F0F1-type ATP synthetase |
| ACT57088 | C | COG0055 | subunit beta of F0F1-type ATP synthetase |
| ACT57087 | C | COG0355 | subunit epsilon of F0F1-type ATP synthetase |
|  |  |  |  |
| ACT57040 | L | COG0593 | ATPase involved in DNA replication |
| ACT57041 | S | unknown | unknown |
| ACT57042 | F | COG0150 | *purM* for 5’-phosphoribosyl-5-aminoimidazole synthetase |
| ACT57043 | F | COG0299 | *purN* phosphoribosylglycinamide formyltransferase |
|  |  |  |  |
| ACT57005 | U | COG1559 | predicted periplasmic solute binding |
| ACT57006 | I | COG0304 | 3-oxoacyl-(acyl carrier protein) synthase II |
| ACT57007 | I | COG0236 | acyl-carrier protein |
| ACT57008 | R | COG1028 | dehydrogenase related to short chain alcohols |
| ACT57009 | I | COG0331 | ACP=s-malonyltransferase |
|  |  |  |  |
| ACT57125 | G | COG3588 | fructose-biphosphate aldolase |
| ACT57126 | G | COG0126 | phosphoglycerate kinase |
| ACT57127 | G | COG0057 | glyceraldehyde3-phosphatedehydodrogense |
| ACT57128 | G | COG0021 | transketolase |
|  |  |  |  |
| ACT56969 | L | COG0776 | bacterial nucleoid binding protein |
| ACT56970 | I | COG0332 | 3-oxoacyl-[acyl-carrier-protein] reductase |
| ACT56971 | I | COG0416 | glycerol-3-phosphate acyltransferase involved with fatty acid/ phospholipid synthesis |
|  |  |  |  |
| ACT57364 | T | COG5002 | Signal transduction histidine kinase |
| ACT57365 | T | COG0784 | two component sensor regulator ; FOG: CheY-like receiver |
| ACT57366 | T | COG0745 | CheY-like receiver domain; winged-helix DNA-binding domain |
| ACT57367 | S | unknown | hypothetical protein |
|  |  |  |  |
| ACT57325 | C | COG0838 | subunit A of NADH ubiquinone oxidoreductase |
| ACT57326 | C | COG0377 | subunit B of NADH ubiquinone oxidoreductase |
| ACT57327 | C | COG0852 | subunit C of NADH ubiquinone oxidoreductase |
| ACT57328 | C | COG0649 | subunit D of NADH ubiquinone oxidoreductase |
| ACT57329 | C | COG1905 | subunit E of NADH ubiquinone oxidoreductase |
|  |  |  |  |
| ACT57330 | S |  | Hypothetical protein |
| ACT57331 | C | COG1894 | subunit F NADH ubiquinone oxidoreductase |
| ACT57332 | C | COG1034 | subunit G of NADH ubiquinone oxidoreductase |
| ACT57333 | C | COG1005 | subunit H of NADH ubiquinone oxidoreductase |
| ACT57334 | C | COG1143 | subunit I of NADH ubiquinone oxidoreductase |
| ACT57335 | C | COG0839 | subunit J of NADH ubiquinone oxidoreductase |
| ACT57336 | S |  | hypothetical protein |
| ACT57337 | C | COG0713 | NADH ubiquinone oxidoreductase chain K |
| ACT57338 | C | COG1009 | NADH ubiquinone oxidoreductase chain L |
| ACT57339 | C | COG1008 | NADH ubiquinone oxidoreductase chain M |
| ACT57340 | C | COG1107 | NADH ubiquinone oxidoreductase chain N |
| ACT57341 | H | COG0340 | biotin-protein ligase *birA* |
| ACT57342 | D | COG0595 | beta-lactamase domain protein |
| ACT57343 | J | COG0442 | prolyl-tRNA synthetase |
| ACT57344 | V | COG1136 | ATPase component of an antimicrobial lipoprotein releasing system |
|  |  |  |  |
| ACT57410 | L | COG1758 | DNA-directed RNA polymerase omega subunit |
| ACT57411 | I | COG0736 | 4'-a phosphopantetheinyltransferase |
| ACT57412 | O | COG0881 | type 1 signal peptidase |
| ACT57413 | L | COG0571 | ribonuclease III |
| ACT57414 | J | COG1159 | GTP-binding Era homolog protein |
| ACT57415 | M | COG1207 | UDP-N-acetylglucosamine 1 phosphate uridyl transferase |
| ACT57416 | G | COG0449 | glucosamine--fructose-6-phosphate amino-transferase |
| ACT57417 | S | COG2938 | uncharacterized conserved hypothetical protein |
| ACT57418 | L | COG1200 | ATP-dependent DNA helicase RecG |
| ACT57419 | S | COG2938 | uncharacterized conserved hypothetical protein |
| ACT57420 | K | COG1197 | transcription repair coupling factor superfamily II helicase |
|  |  |  |  |
| ACT57432 | M | COG0330 | putative hydrolase serine protease transmembrane |
| ACT57431 | M | COG0330 | membrane hflK-like protease |
| ACT 57430 | H | COG0262 | dihydrofolate reductase |
| ACT 57429 | F | COG3814 | thymidylate synthase |
|  |  |  |  |
| ACT57452 | J | COG0064 | aspartyl/ glutamyl-tRNA amidotransferase (but not in *Bartonella henselae*) |
| ACT57451 | J | COG0154 | aspartyl/glutamyl-tRNA amidotransferase subunit |
| ACT57450 | J | COG0721 | glutamyl-tRNA(Gln) amidotransferase |
| ACT57449 | L | COG0816 | Holliday junction resolvase, YqgF |
|  |  |  |  |
|  |  |  |  |
| ACT57524 | I | COG0567 | 2-oxoglutarate dehydrogenase/dehydrogenase E1 component |
| ACT57525 | I | COG0508 | pyruvate 2-oxoglutarate dehydrogenase/dihydro lipoamide acyl transferase E2 component |
| ACT57526 | I | COG1249 | pyruvate 2-oxoglutarate dehydrogenase/dihydro lipoamide dehyfrogenase E3 component |
|  |  |  |  |
| ACT57506 | M | COG0457 | HemY domain-containing protein |
| ACT57507 | S | COG4223 | uncharacterized conserved hypothetical protein |
| ACT 57511 | M | COG0533 | metal dependent protease; possible chaperone activity/ o-sialoglycoprotein endopeptidase |
|  |  |  |  |
| ACT57578 | M | COG0275 | SAM-dependent methyltransferase MraW involved in cell envelope development |
| ACT57577 | U | COG5462 | Predicted secreted (periplasmic) protein |
| ACT57576 | M | COG0768 | penicillin-binding protein involved in peptidoglycan synthesis |
| ACT57575 | M | COG0769 | UDP-N-acetylmuramoylalanyl-D-glutamate2,6-diaminopimelate ligase |
| ACT57574 | M | COG0770 | UDP-N-acetylmuramoylalanyl-D-glutamate ligase |
| ACT57573 | M | COG0472 | phospho-N-acetylmuramoyl-pentapeptide transferase |
| ACT57572 | M | COG0771 | UDP-N-acetylmuramoyl-L-alanyl-D-glutamate synthetase |
| ACT57571 | M | COG0772 | ftsW cell division protein |
| ACT57570 | M | COG0707 | N-acetylglucosaminyl transferase |
| ACT57569 | M | COG0773 | UDP-N-acetylmuramate-L-alanine ligase |
| ACT57568 | M | COG0812 | UDP-N-acetylenolpyruvoylglucosamine reductase |
|  |  |  |  |
| ACT57514 | C | COG2009 | subunit of succinate/fumerate dehydrogense |
| ACT57515 | C | COG2142 | subunit of succinate/fumerate dehydrogense |
| ACT57516 | C | COG1053 | subunit of succinate/fumerate dehydrogense |
| ACT57517 | C | COG0479 | subunit of succinate/fumerate dehydrogense |
|  |  |  |  |
| ACT57532 | P | COG0719 | ABC Fe-S cluster assembly transport SufB cysteine desulfurase |
| ACT57533 | P | COG0396 | SufC FeS assembly ATPase |
| ACT57534 | P | COG0719 | SufD FeS assembly ATPase |
| ACT57535 | P | COG0520 | SufS-like putative aminotransferase |
| ACT57536 | P | COG2151 | FeS assembly Suf iron-sulfur cluster biosynthetic transport protein |
|  |  |  |  |
| ACT57561 | C | COG0356 | subunit of H^+^-transporting proton pump two-sector F0F1-type ATPase/ ATP synthetase |
| ACT57560 | C | COG0636 | subunit of H^+^-transporting proton pump two-sector F0F1-type ATPase/ ATP synthetase |
| ACT57559 | C | COG0711 | subunit of H^+^-transporting proton pump two-sector F0F1-type ATPase/ ATP synthetase |
| ACT57558 | C | COG0711 | subunit of H^+^-transporting proton pump two-sector F0F1-type ATPase/ ATP synthetase |
|  |  |  |  |
| ACT57645 | L | COG0272 | NAD-dependent DNA ligase-contains BRCT domain type II |
| ACT57646 | L | COG0497 | ATPase involved in DNA repair |
| ACT57647 | R | COG4105 | DNA uptake lipoprotein |
| ACT57648 | D | COG0774 | UDP-3-O-acyl-N-acetylglucosamine deacetylase |
| ACT57649 | D | COG0206 | cell division GTPase |
| ACT57650 | D | COG0849 | actin-like ATPase involved in cell division |
| ACT57651 | D | COG1589 | cell division septal protein |
|  |  |  |  |

^a^ Protein accession number in GenBank. ^b^ Functional class and category (Konstantinidis and Teidje 2004). ^c^ Cluster of Orthologous Groups of proteins. The protein IDs are highlighted to designate assignment to COG functional classes: red for information storage and processing, green for cellular processes and yellow for metabolism.
