# Supplementary material for: Conservation of Gene Order and Content in the Circular Chromosomes of ‘Candidatus Liberibacter asiaticus’ and Other Rhizobiales
Source: PLoS One. 2012 Apr 4;7(4):e34673. doi: 10.1371/journal.pone.0034673 (PMC3319617; doi:10.1371/journal.pone.0034673)
Supplement: Table S2 — Proteins annotated from the ‘ Ca. Liberibacter asiaticus circular chromosome and shared with only one other member of the Rhizobiales studied B. henselae , S. meliloti , A. tumefaciens , B. japonicum . (DOCX) [file pone.0034673.s004.docx]

**Table S2.** Proteins annotated from the ‘*Ca*. Liberibacter asiaticus circular chromosome and shared with only one other member of the Rhizobiales studied *B. henselae, S. meliloti*, *A. tumefaciens*, *B. japonicum*.

| **Protein Accession ID** | | **E-value** | **Annotation in *Bartonella henselae*** |
| --- | --- | --- | --- |
| ACT56607 | CAF27486 | 2.00E-55 | Integrase/recombinase |
| ACT56764 | CAF28082 | 1.00E-06 | hypothetical protein |
| ACT56811 | CAF28183 | 6.00E-25 | hypothetical protein |
| ACT56835 | CAF27213 | 5.00E-48 | Na+/H antiporter |
| ACT57214 | CAF28409 | 1.00E-122 | Carboxynorspermidine decarboxylase |
| ACT57215 | CAF28410 | 1.00E-149 | hypothetical protein |
| ACT57529 | CAF28224 | 2.00E-25 | Phage-related lysozyme |
| ACT57530 | CAF27719 | 4.00E-07 | Phage related lysozyme |
| ACT57531 | CAF28224 | 5.00E-10 | phage related lysozyme |
| ACT57604 | CAF27726 | 2.00E-06 | hypothetical genomic island protein |
| ACT57695 | CAF27492 | 4.00E-07 | phage related protein |
| ACT57696 | CAF27492 | 7.00E-07 | phage related protein |
|  |  |  | **Annotation in *Sinorhizobium meliloti*** |
| ACT56861 | CAC47421 | 1.00E-15 | Hypothetical protein |
| ACT57022 | CAC47319 | 6.00E-12 | Hypothetical signal peptide protein |
| ACT57034 | CAC47831 | 5.00E-87 | Thiamine-binding periplasmic protein precursor |
| ACT57071 | CAC46264 | 5.00E-07 | Probable outer-membrane protein |
| ACT57276 | CAC47911 | 3.00E-08 | Conserved hypothetical protein |
| ACT57306 | CAC47421 | 3.00E-12 | Hypothetical protein |
| ACT57407 | CAC47421 | 1.00E-09 | Hypothetical protein |
| ACT57488 | CAC45575 | 1.00E-06 | Hypothetical protein |
| ACT57581 | CAC47421 | 1.00E-12 | Hypothetical protein |
| ACT57583 | CAC47421 | 3.00E-13 | Hypothetical protein |
| ACT57619 | CAC46599 | 2.00E-10 | Hypothetical protein |
|  |  |  | **Annotation in *Agrobacterium tumefaciens*** |
| ACT56967 | AAK86800 | 1.00E-64 | cation efflux system component |
| ACT57036 | AAK86222 | 1.00E-06 | ABC transporter, substrate binding protein (iron) |
| ACT57209 | AAK86042 | 2.00E-05 | component of type IV pili, a pilin subunit |
| ACT57371 | AAK87028 | 5.00E-37 | hypothetical protein Atu8150 |
| ACT57587 | AAK87569 | 3.00E-12 | conserved hypothetical protein |
|  |  |  | **Annotation in *Bradyrhizobium japonicum*** |
| ACT56609 | BAC45271 | 7.00E-08 | ribonucleoside-diphosphate reductase beta subunit |
| ACT56675 | BAC47748 | 2.00E-81 | bll2483 |
| ACT56857 | BAC48979 | 4.00E-06 | bll3714 |
| ACT56958 | BAC47360 | 1.00E-103 | biotin synthetase |
| ACT56960 | BAC47363 | 5.00E-59 | dethiobiotin synthase |
| ACT57134 | BAC45333 | 7.00E-30 | exonuclease I |
| ACT57479 | BAC45271 | 7.00E-08 | ribonucleoside-diphosphate reductase beta subunit |
| ACT57504 | BAC49760 | 4.00E-84 | blr4495 |
| ACT57505 | BAC47747 | 1.00E-15 | bll2482 |
| ACT57663 | BAC53524 | 2.00E-11 | bll8259 |
| ACT57698 | BAC53522 | 9.00E-24 | bll8257 |

^a^ Data are the accession ID’s of the genes encoding an orthologous protein pair in ‘*Ca*. Liberibacter asiaticus’ and in the other bacterial species, and the E-value of the BlastP result between orthologous proteins in ‘*Ca*. Liberibacter asiaticus’ and in the other member of the Rhizobiales, and the resulting annotation.
