# Supplementary material for: Conservation of Gene Order and Content in the Circular Chromosomes of ‘Candidatus Liberibacter asiaticus’ and Other Rhizobiales
Source: PLoS One. 2012 Apr 4;7(4):e34673. doi: 10.1371/journal.pone.0034673 (PMC3319617; doi:10.1371/journal.pone.0034673)
Supplement: Table S3 — Proteins encoded by the ‘ Ca. Liberibacter asiaticus chromosome that did not have orthologs in S. meliloti , A. tumefaciens , B. japonicum and B. henselae . (DOCX) [file pone.0034673.s005.docx]

**Table S3. P**roteins encoded by the ‘*Ca*. Liberibacter asiaticus chromosome that did not have orthologs in *S. meliloti*, *A. tumefaciens*, *B. japonicum* and *B. henselae*.

| **Accession** | **‘Ca. Liberibacter asiaticus’** | **Pfam or CCD results**^a^ | **Pfam or CCD Domain** | **E-value** |
| --- | --- | --- | --- | --- |
|  |  |  |  |  |
|  |  | **Information Storage and Processing** |  |  |
|  |  |  |  |  |
| ACT56601 | VRR-NUC domain protein | VRR-NUC domain protein | VRR_NUC | 1.30 e-15 |
| ACT56602 | hypothetical protein | SNF2-related Dead box helicase | DEX_Dc | 2.91 e-14 |
| ACT56603 | hypothetical protein | DNA/RNA helicases, SNF2 family | HepA | 1.01 e-07 |
| ACT56674 | Hypothetical protein | Lipopolysaccharide biosynthesis | RgpF | 9.90 e-28 |
| ACT57037 | Hypothetical protein | The substrate binding domain of LysR-typ) | LTTR | 3.28 e-04 |
| ACT57462 | GCN5-related N-acetyltransferase | N-Acyltransferase superfamily | Nat_SF | 5.08 e-07 |
| ACT57468 | Endo/exonuclease/phosphatase | Endo/exonuclease/phosphatase | MnuA | 4.65 e-03 |
| ACT57476 | hypothetical protein | Endo/exonuclease/phosphatase | Exo_endo_phos | 3.70 e-13 |
| ACT57620 | transcriptional regulator^b^ | transcriptional regulator | HTH_XRE | 6.40 e-08 |
| ACT57621 | hypothetical protein | replicative DNA helicase | DnaB | 2.11 e-05 |
| ACT57623 | hypothetical protein | replicative DNA helicase | DnaB | 9.96 e-05 |
| ACT57664 | hypothetical protein | Clustered Reg Interspaced Short Palindrome | Cas4 | 3.19 e-29 |
| ACT57667 | hypothetical protein | VRR-NUC domain protein | VRR_NUC | 2.60 e-06 |
| ACT57668 | SNF2 related | SNF2 related | DEXDc | 4.70 e-09 |
| ACT57671 | Guanylate kinase | none | none | none |
| ACT57679 | hypothetical protein | Chromosome segregation protein, provisional | PRK02224 | 5.90 e-03 |
| ACT57690 | hypothetical protein | Transposase | Transposase_8 | 1.40 e-06 |
|  |  |  |  |  |
|  |  | **Cellular Processes** |  |  |
|  |  |  |  |  |
| ACT56942 | zinc-binding protein | zinc-binding protein | DUF329 | 2.50 e-06 |
| ACT56987 | type II restriction endonuclease | None | None |  |
| ACT56988 | hypothetical | None | None |  |
| ACT56989 | type II modification methyltransferase | C-5 cytosine-specific DNA methylase | Cyt_C5_DNA_methylase | 5.39e-30 |
| ACT56990 | DNA-methyltransferase MKpn2kI | C-5 cytosine-specific DNA methylase | DNA_methylase | 2.90 e-21 |
| ACT57113 | Hypothetical protein | P-loop_NTPase super family | P-loop NTPase | 2.97 e-09 |
| ACT57193 | Ferroxidase | Ferroxidase | Ferritin | 1.50 e-24 |
| ACT57196 | hypothetical protein | pilus assembly protein | transmembrane |  |
| ACT57205 | hypothetical protein | Flp/Fap pilin component | Flp_Fap | 4.50 e-19 |
| ACT57206 | hypothetical protein | Flp/Fap pilin component | Flp_Fap | 3.50 e-20 |
| ACT57310 | putative restriction endonuclease S | res/mod system DNA specificity subunit | Methylase_S | 5.90 e-05 |
| ACT57316 | glucose/galactose transporter | glucose/galactose transporter | MFS_1 | 6.30 e-14 |
| ACT57391 | glycosyl transferase family protein | glycosyl transferase family protein | Glycosyl_trans_2 | 3.50 e-28 |
| ACT57392 | dTDP-4-dehydrorhamnose 3,5-epim | dTDP-4-dehydrorhamnose 3,5-epim | dTDP_sugar_isom | 1.50 e-86 |
| ACT57394 | dTDP-4-dehydrorhamnose reductase | dTDP-4-dehydrorhamnose reductase | RmID_sub_bind | 1.80e-115 |
| ACT57396 | Hypothetical protein | Lipopolysaccharide biosynthesis protein | RgpF | 3.20 e-38 |
| ACT57655 | Hypothetical protein | type I restriction enzyme EcoKI subunit R | HsdR | 1.36 e-05 |
|  |  |  |  |  |
|  |  | **Metabolism** |  |  |
|  |  |  |  |  |
| ACT56796 | ATP/ADP translocase | ATP/ADP translocase | TLC | 4.40e-241 |
| ACT56857 | serralysin | serralysin | ZnMc | 3.48 e-04 |
| ACT57036 | extracellular solute-binding protein | extracellular solute-binding protein | N/A |  |
| ACT57165 | hypothetical protein | Acyl-CoA dehydrogenase | ACAD Superfamily | 2.21 e -03 |
| ACT57489 | diphosphomevalonate decarboxylase | diphosphomevalonate decarboxylase | GHMP_kinases_N | 1.70 e-11 |
| ACT57490 | GHMP kinase | GHMP kinase | GHMP_kinases_N | 2.10 e-10 |
| ACT57491 | GHMP kinase | GHMP kinase | GHMP_kinases_N | 5.70 e-11 |
| ACT57492 | hydroxymethylglutaryl-coA synthase | hydroxymethylglutaryl-coA synthase | HMG_CoA_synt_N | 8.20 e-17 |
| ACT57493 | isopentenyl pyrophosphate isomerase | isopentenyl pyrophosphate isomerase | FMN_dh | 1.80 e-04 |
| ACT57494 | HMG-CoA reductase | HMG-CoA reductase | HMG-CoA_red | 6.40 e-33 |
| ACT57495 | hypothetical protein | Inner membrane protein ydjM | DUF457 | 1.40 e-21 |
| ACT57543 | Hypothetical protein | Beta glucosidase related glycosidases | GglX | 1.23 e-04 |
| ACT57544 | Glycosyl hydrolase family protein | None | None | None |
| ACT57624 | hypothetical | unknown function 95 residues in length | DUF1376 | 6.06 e-05 |
| ACT57625 | hypothetical | Unknown function 95 residues in length | DUF1376 | 4.94 e-12 |
| ACT57642 | ascorbate PTS system enzyme IIC/IIB | ascorbate PTS system enzyme IIC/IIB | SgaT_UlaA | 6.30e-171 |
| ACT57671 | guanylate kinase | guanylate kinase | transmembrane |  |
|  |  |  |  |  |

^a^ Proteins as annotated from the published sequence of the ‘*Ca*. Liberibacter asiaticus’ circular chromosome and from protein BLAST against the pFAM and CCD databases. The better match of the two is presented.

^b^ Proteins highlighted in gray had a similar protein in one of the other species, but the e-value was weak and the annotations for the similar proteins differed.

^c^ The proteins are listed by COG functional groups (Konstantinidis and Teidje 2004)
