# Supplementary material for: Conservation of Gene Order and Content in the Circular Chromosomes of ‘Candidatus Liberibacter asiaticus’ and Other Rhizobiales
Source: PLoS One. 2012 Apr 4;7(4):e34673. doi: 10.1371/journal.pone.0034673 (PMC3319617; doi:10.1371/journal.pone.0034673)
Supplement: Table S4 — Proteins encoded by the ‘ Ca. Liberibacter asiaticus’ chromosome that did not have orthologs in S. meliloti , A tumefaciens , B. japonicum and B. henselae and which encode phage related genes. (DOCX) [file pone.0034673.s006.docx]

**Table S4**. Proteins encoded by the ‘*Ca*. Liberibacter asiaticus’ chromosome that did not have orthologs in *S. meliloti, A tumefaciens, B. japonicum* and *B. henselae* and which encode phage related genes.

| **Accession** | **‘Ca. Liberibacter asiaticus’** | **Pfam or CCD results** | **Pfam or CCD Domains** | **e-value** |
| --- | --- | --- | --- | --- |
|  |  |  |  |  |
| ACT57028 | hypothetical protein | Prophage antirepressor (transcription) | Prophage antirepressor | 4.98e-05 |
| ACT57486 | phage-associated protein | phage-associated protein | GepA | 2.10 e-16 |
| ACT57530 | Hypothetical protein | Endolysins and autolysins | COG3772 | 9.42 e-12 |
| ACT56597 | hypothetical protein | Clustered Regularly Interspaced Short Palindromes | Cas4 | 4.70 e-79 |
| ACT56598 | prophage antirepressor | BRO family, N-terminal domain | Bro-N | 1.35E-33 |
| ACT56599 | hypothetical protein | Phage protein unknown function | DUF2815 | 4.28 e-25 |
| ACT57660 | Putative phage terminase | Terminase-like family | Terminase_6 | 9.80 e-03 |
| ACT57665 | hypothetical protein | phage related unknown function | DUF2815 | 5.34 e-35 |
| ACT57675 | hypothetical protein | Phage Structural protein | PHA01972 | 1.65 e-57 |
| ACT57681 | hypothetical protein | Phage hypothetical protein | PHA00661 | 1.63 e-30 |
| ACT57686 | head-to-tail joining protein, put | head-to-tail joining protein | Head-tail_con | 4.40 e-70 |
| ACT57688 | putative phage terminase | Terminase like family | Terminase_6 | 2.19 e-10 |

^a^ Proteins as annotated from the published sequence of the ‘*Ca*. Liberibacter asiaticus’ circular chromosome and results from protein BLAST against the pFAM and CCD databases. The better match of the two is presented.
